# Systemic Lupus Erythematosus With Cardiac Tamponade and Myocardial Edema in the Early Postpartum Period

**DOI:** 10.1016/j.jaccas.2025.104399

**Published:** 2025-07-30

**Authors:** Asad Shabbir, Fatima A.M. Abdulsalam, Sam Dawkins, Marco Spartera, Henry Boardman, Alexandra E. Cairns, Antonios Psarras, Elizabeth Orchard, Rohan Wijesurendra

**Affiliations:** a John Radcliffe Hospital, Oxford University Hospital Trust, Oxford, United Kingdom; b Kennedy Institute of Rheumatology, NDORMS, University of Oxford, Oxford, United Kingdom; c Rheumatology Department, Nuffield Orthopaedic Centre, Oxford University Hospital Trust, Oxford, United Kingdom; d University of Oxford, Oxford, United Kingdom

**Keywords:** pericardial effusion, pregnancy, tamponade

## Abstract

**Background:**

Pericardial effusions are frequently caused by inflammatory diseases. In cases of serosal inflammation, which often present with concomitant systemic symptoms, cardiac tamponade can occur, requiring emergency drainage. Nevertheless, it is rare for the index presentation of a previously undiagnosed inflammatory disease to be with cardiac tamponade.

**Case Summary:**

We describe a case of a young woman who presented in the postpartum period with cardiac tamponade. Further investigations confirmed that the underlying diagnosis was systemic lupus erythematosus (SLE).

**Discussion:**

SLE can be associated with pericardial effusion but rarely causes cardiac tamponade. Herein, we describe a case of an index presentation of SLE in the postpartum period with a large pericardial effusion and tamponade. Cardiac imaging showed myocardial edema, reflective of associated myocarditis.

**Take-Home Messages:**

SLE can present in the immediate postpartum period, and acute management of tamponade in this context includes drainage of the effusion and immunosuppressive therapy.

## Visual Summary

**Figure:**
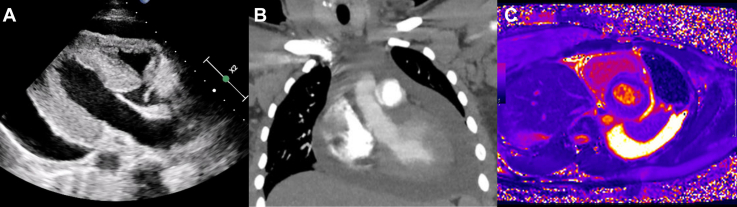

## History of Presentation

A 35-year-old woman attended her local emergency department with breathlessness, which had progressively worsened since giving birth to her first child 2 weeks previously. She denied chest pain, palpitation, syncope, or other constitutional symptoms.

**Take-Home Messages**

• The management of systemic lupus erythematosus–induced tamponade in the postpartum period is the same as that in patients who were not recently pregnant, with emergency drainage of the effusion and the initiation of immunosuppressive therapy.
• When managing systemic lupus erythematosus in pregnancy, consideration should be given to both safe medication choices and, where anti-Ro/La antibodies are positive, additional fetal monitoring due to the risk of congenital heart block.

## Past Medical History

An urgent cesarean delivery had been undertaken at 35 + 6 weeks of gestation because of intrauterine growth restriction. The patient was a sickle cell gene carrier and had no other medical history.

## Differential Diagnoses

In the early phase of the admission, the differential diagnoses included pulmonary embolus, community-acquired chest infection, or a peripartum cardiomyopathy.

## Investigations

**Figure 1:**
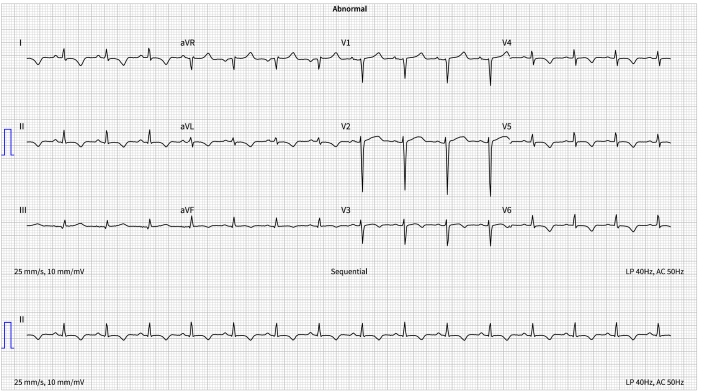
12-Lead Electrocardiogram 12-lead electrocardiogram showing sinus rhythm and inferior-lateral T-wave inversion.

**Figure 2:**
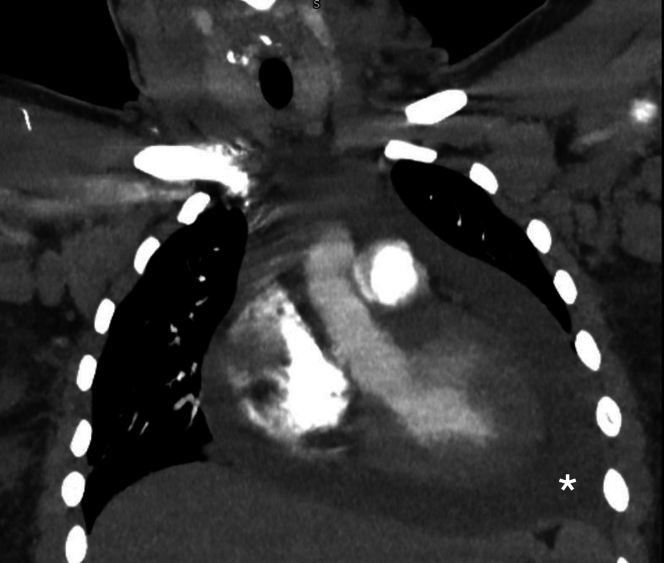
CT Pulmonary Angiogram The CT pulmonary angiogram excluded pulmonary embolism but did confirm a 3-cm pericardial effusion (asterisks), pulmonary edema, contrast reflux into the inferior vena cava, and diffuse lymphadenopathy. CT = computed tomography.

Notable findings on clinical assessment included tachycardia (120 beats/min), tachypnea (32 breaths/min), muffled heart sounds, elevated Jugular venous pressure, and pyrexia of 38.8 °C. Blood pressure was maintained at 130/80 mm Hg. The 12-lead electrocardiogram showed inferior-lateral T-wave inversion in the absence of chest pain (Figure 1). Notable clinical hematology and biochemistry findings included a hemoglobin level of 92 g/L, a C-reactive protein (CRP) level of 115 mg/L, a high-sensitivity troponin I level of 261 ng/L, and a creatinine level of 80 μmol/L. A computed tomography pulmonary angiogram excluded pulmonary embolism and confirmed a large pericardial effusion with a depth of 3 cm (Figure 2), evidence of pulmonary edema, contrast reflux into the inferior vena cava, and diffuse lymphadenopathy. An emergency transfer to our institution was arranged.

**Figure 3:**
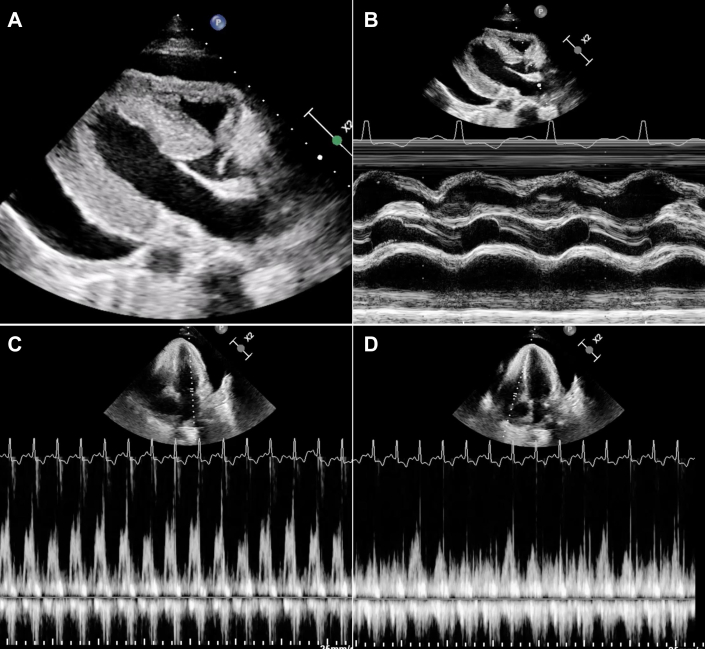
Transthoracic Echocardiogram Echocardiography demonstrated a large pericardial effusion on 2-dimensional imaging (A), with right ventricular diastolic collapse identified on M-mode imaging (B), and significant variation of mitral (C) and tricuspid valve inflow (D) on pulse-wave imaging.

We undertook an emergency transthoracic echocardiogram that confirmed a large pericardial effusion (Figure 3A, Video 1), with echocardiographic features of cardiac tamponade including right ventricular diastolic collapse (Figure 3B) and significant respiratory variation of mitral valve and tricuspid valve inflow (Figures 3C and 3D, respectively). There was also the appearance of marked left ventricular hypertrophy (up to 20 mm), with preserved left ventricular systolic function.

## Management

The patient underwent emergency pericardiocentesis, and approximately 700 mL of straw-colored gelatinous liquid was drained (sent for culture, protein, acid-fast bacilli, cell count, and lactate dehydrogenase), with an immediate improvement in hemodynamics and symptoms.

**Figure 4:**
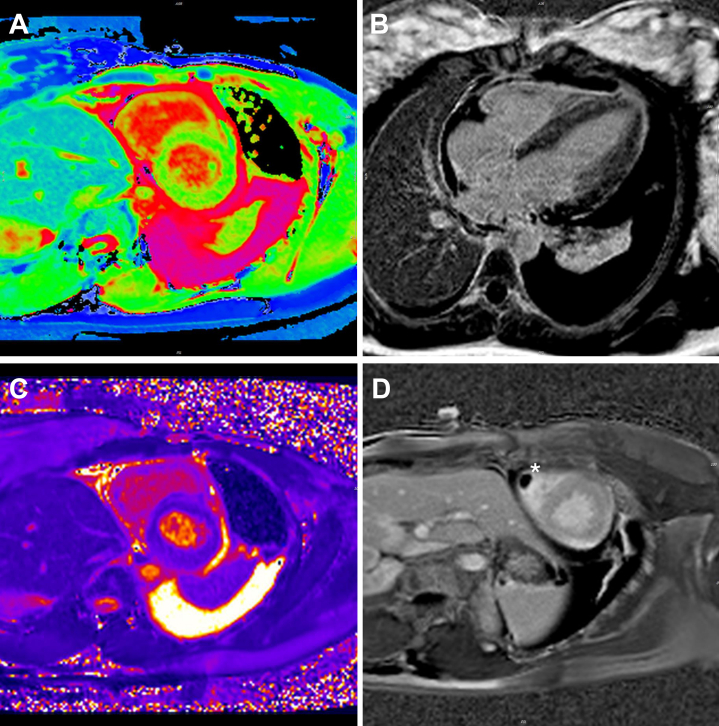
Cardiac Magnetic Resonance ShMOLLI T1 map showing globally elevated myocardial T1 values and pericardial and pleural effusions (A), PSIR imaging showing patchy high signal intensity in the myocardium consistent with global edema (B), T2 map showing globally elevated myocardial T2 values (C), and early gadolinium imaging showing the presence of a right ventricular thrombus (D; asterisk). PSIR = phase-sensitive inversion recovery; ShMOLLI = Shortened MOdified Look-Locker Inversion recovery.

Once the pericardial drain had been removed, a cardiac magnetic resonance (CMR) scan was organized and identified globally elevated T1 and T2 signal suggestive of very marked myocardial edema with a reduction in the degree of left ventricular hypertrophy compared with the earlier echocardiogram (Figures 4A to 4C, Supplemental Figures 1 and 2), consistent with active myocarditis. Furthermore, a 13 × 7 mm thrombus was present at the apex of the right ventricle (Figure 4D). Left ventricular, right ventricular, and valvular function were normal. A small-moderate pericardial effusion and large left pleural effusion were noted.

Given the ventricular thrombus, anticoagulation was initiated with low-molecular-weight heparin. The patient experienced persistent fever in the absence of any positive cultures (pericardium, blood, and urine), prompting the suspicion of an underlying systemic cause for the presentation. An autoimmune screen was positive with antinuclear antibody >640, and positive anti-Ro, anti-La, and double-stranded DNA titers, consistent with a diagnosis of systemic lupus erythematosus (SLE). A comprehensive list of related lab work is provided in Supplemental Table 1.

A rheumatology opinion was sought, and a diagnosis of SLE was confirmed. The patient was initiated on azathioprine, hydroxychloroquine, and prednisolone, with immediate resolution of the previously persistent pyrexia. Azathioprine and hydroxychloroquine were initiated owing to the patient's desire for future pregnancies and breastfeeding. Cyclophosphamide was avoided as the troponin was down-trending and the CRP normalized. The patient was discharged from hospital shortly afterward and remains well at follow-up with no recurrence of pericardial effusion, and no other abnormality noted on repeat transthoracic echocardiography at 4 weeks.

## Discussion

Systemic autoimmune conditions are common causes of pericardial effusion.^1^ Examples of such diseases include rheumatoid arthritis, SLE, Sjögren syndrome, and mixed connective tissue disease.^2^ These disorders often present with pyrexia, fatigue, arthritis, and cutaneous lesions, among other symptoms. Some patients can experience chest pain with myopericarditis, which is associated with pericardial effusion.^3^ Rarely, cardiac tamponade has been described as the index presentation of a systemic inflammatory disease in the absence of other symptoms.^4^ Pregnancy and the postpartum period are associated with a marked immune shift and a concomitant risk of acute flares of systemic inflammatory diseases.^5^ Pregnancy outcomes in SLE are worse, and postpartum flares are often seen with high disease activity.^6^

We present a case of a 35-year-old female patient who attended hospital with subacute breathlessness in the postpartum period, having previously been fit and well. She was found to have a large pericardial effusion with tamponade physiology. After emergency pericardial drainage, she experienced ongoing pyrexia, prompting a heightened suspicion of an underlying systemic inflammatory condition as the underlying unifying diagnosis. A diagnosis of SLE was made after the confirmation of positive SLE serology; the presence of active serositis, myocarditis, and cutaneous lesions; and consistent CMR. This case highlights the importance of prompt and appropriate recognition and management of cardiac tamponade with emergency pericardiocentesis, the need to consider a wide differential diagnosis when faced with a scenario of unexplained persistent pyrexia, and ensuring precautions for breastfeeding mothers and investigating for possible cardiac disease in the newborn child.

One of the commonest causes of postpartum pericardial effusions is myopericarditis, which usually presents with symptoms of chest pain. In our case, the presenting symptom was breathlessness without chest pain, accompanied by physical signs suggestive of cardiac tamponade. Emergency bedside transthoracic echocardiography confirmed the diagnosis, highlighting its utility in assessing patients with clinical features of cardiac tamponade, even in the absence of chest pain.

Although SLE is associated with pericardial effusions and can present with tamponade in patients with known disease,^7^ cardiac tamponade as the index presentation of SLE is rare, and very few cases have been reported in the literature.^4^^,^^8^^,^^9^ The dramatic presentation in our case with tamponade could be, in part, related to a predominant serositis, reflected by the very raised CRP, which would normally be expected to be only moderately raised in lupus flares. This is also supported by evidence of serosal inflammation of the lungs with bilateral pleural effusions.

In this case, the diagnosis of myocarditis was secured with CMR, and specifically by very high T1 and T2 values on parametric mapping, highlighting the role of CMR in tissue characterization and the ability to differentiate causes of left ventricular hypertrophy. This, along with ongoing pyrexia, suggested a diagnosis other than simple pericarditis and prompted heightened suspicion of an underlying systemic inflammatory disease.^10^ Given the prompt normalization of the echocardiographic appearances after treatment for SLE, repeat CMR was not undertaken but can be considered if there is any future concern over cardiac involvement of SLE.

We believe that the finding of the right ventricular thrombus was reflective of active myocarditis. Given that the patient sought to continue breastfeeding, we chose to continue a higher dose of low-molecular-weight heparin, rather than transition to a direct oral anticoagulant or warfarin. Furthermore, with the effective anti-inflammatory treatment for the SLE, a prolonged course of anticoagulation was not considered to be required, and therefore, we chose to limit the duration of anticoagulation to 3 months in total.

In the postpartum period, the immune profile of the mother changes. Some data quantifying the risks of flares in patients with known systemic inflammatory diseases in the postpartum period suggest that there is an increased risk of flare.^11^ Case descriptions of index presentations of SLE in the postpartum period are rare; we are not aware of a previous reported case of an index presentation of SLE in the postpartum period with cardiac tamponade.

## Conclusions

This case emphasizes the value of obtaining expert opinion from the multidisciplinary team, specifically the maternal medicine and rheumatology teams regarding the diagnosis and management of our patient with a dramatic presentation of cardiac tamponade as the index presentation of SLE in the early postpartum period. After emergency pericardiocentesis, our collective opinion was that immunosuppressive therapy should be initiated, with breastfeeding precautions. Furthermore, neonatal screening with electrocardiography and echocardiography was recommended to exclude neonatal cardiac involvement, as the mother was found to be anti-Ro/La positive, with an associated risk of congenital heart block.

**Visual Summary:**
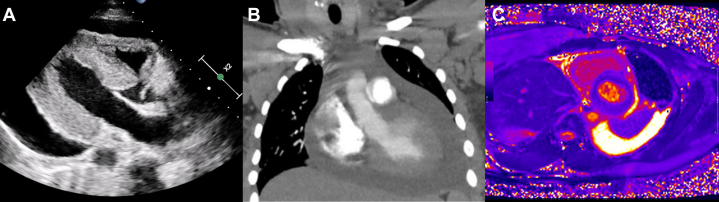
Cardiac Tamponade as Index Presentation of SLE in Postpartum Period A 35-year-old patient presented to the accident and emergency department with symptoms of breathlessness in the postpartum period and was found to be have cardiac tamponade on transthoracic echocardiography (A), and with a large pericardial effusion on computed tomography (B). Later, after pericardiocentesis, the patient underwent a cardiac magnetic resonance that demonstrated myocarditis (C) and was diagnosed with systemic lupus erythematosus. Anti-inflammatory therapy was initiated and the patient made a full recovery.

## Funding Support and Author Disclosures

Dr Wijesurendra has received funding from the 10.13039/100014461National Institute for Health Research Biomedical Research Centre based at Oxford University Hospitals NHS Foundation Trust at the University of Oxford, UK. All other authors have reported that they have no relationships relevant to the contents of this paper to disclose.

## Appendix

Supplemental Material Xxx

## References

[1] Imazio M. (2011). Pericardial involvement in systemic inflammatory diseases. Heart.

[2] Knockaert D.C. (2007). Cardiac involvement in systemic inflammatory diseases. Eur Heart J.

[3] Ramirez G.A., Holopainen N.E.A., Gerosa M. (2025). Distinctive clinical traits of lupus-related myocarditis: a multicentre retrospective study. Rheumatology (Oxford).

[4] Maharaj S.S., Chang S.M. (2015). Cardiac tamponade as the initial presentation of systemic lupus erythematosus: a case report and review of the literature. Pediatr Rheumatol Online J.

[5] Brann E., Edvinsson A., Rostedt Punga A., Sundstrom-Poromaa I., Skalkidou A. (2019). Inflammatory and anti-inflammatory markers in plasma: from late pregnancy to early postpartum. Sci Rep.

[6] Kalok A., Abdul Cader R., Indirayani I. (2019). Pregnancy outcomes in systemtic lupus erythematosus (SLE) in women. Horm Mol Biol Clin Investig.

[7] Bhatt T., Schmidt P., Qasim A., Lajara P., Ganti A., Khaja M. (2023). A 35-year-old female with a lupus flare presenting as cardiac tamponade: a case report. Cureus.

[8] Zhang X., Wu W. (2018). Cardiac tamponade as the initial symptom due to systemic lupus erythematosus in a young man: a case report. Medicine (Baltimore).

[9] Iqbal R., Linn H.N., Gollamudi S. (2025). Cardiac tamponade as initial presentation of SLE: a case report and review of literature. Radiol Case Rep.

[10] Lewis A.J.M., Burrage M.K., Ferreira V.M. (2020). Cardiovascular magnetic resonance imaging for inflammatory heart diseases. Cardiovasc Diagn Ther.

[11] Davis-Porada J., Kim M.Y., Guerra M.M. (2020). Low frequency of flares during pregnancy and post-partum in stable lupus patients. Arthritis Res Ther.

