# Supplementary material for: Systemic Lupus Erythematosus With Cardiac Tamponade and Myocardial Edema in the Early Postpartum Period
Source: JACC Case Rep. 2025 Jul 30;30(21):104399. doi: 10.1016/j.jaccas.2025.104399 (PMC12441525; doi:10.1016/j.jaccas.2025.104399)
Supplement: Supplemental Material — Xxx [file mmc1.docx]

**SUPPLEMENTAL MATERIAL**

**SUPPLEMENTARY FIGURE 1**

Multiview PSIR imaging showing global patchy areas of high signal throughout the myocardium, consistent with myocarditis.

**
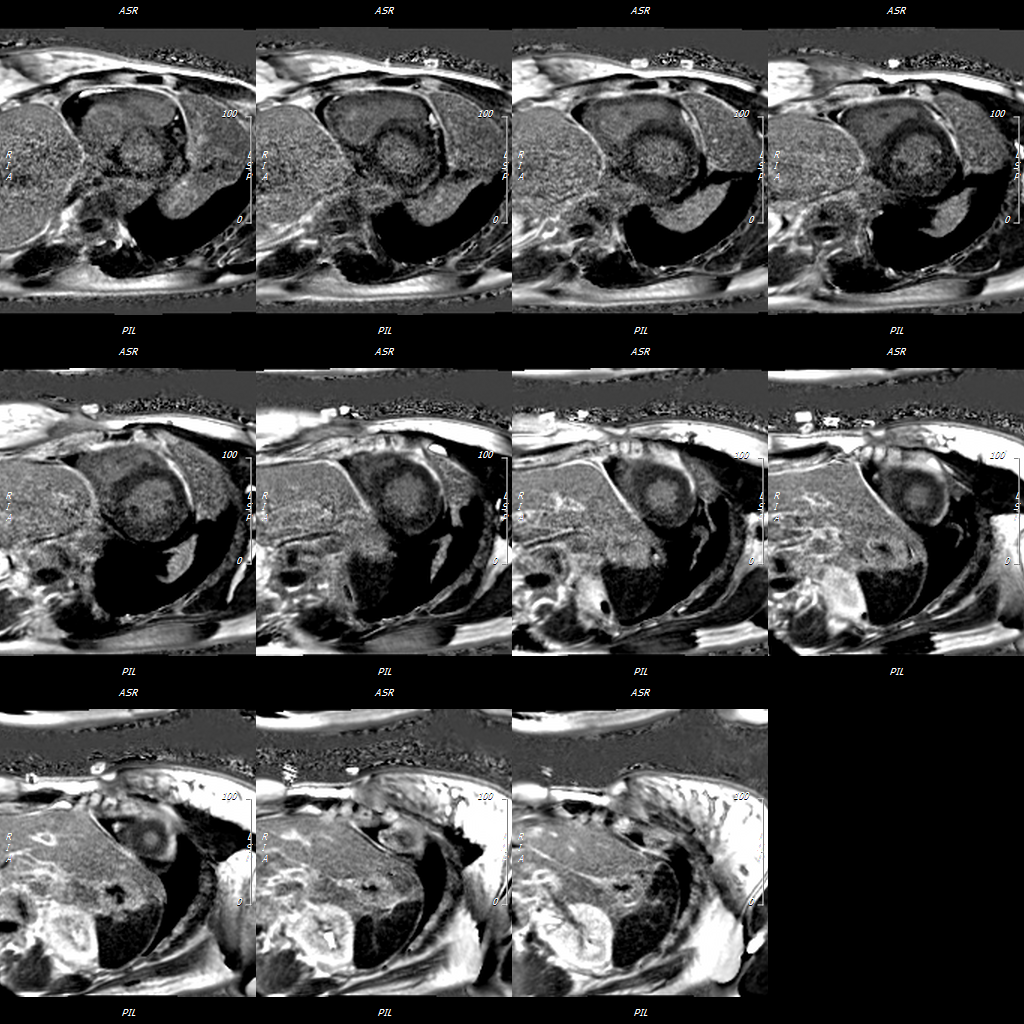
**

**SUPPLEMENTARY FIGURE 2**

AHA 16-segment models showing global elevation in T1 (A) and T2 (B) values. Normal T1 range for females 989ms - 1085ms, normal T2 range for females 50ms – 57ms.

**SUPPLEMENTARY TABLE 1**

| TEST | RESULT | REFERENCE RANGE |
| --- | --- | --- |
| **ANA** | **>640, speckled** | **0-80** |
| **ANCA** | **Positive** | **-** |
| **Anti-La** | **Positive** | **-** |
| Antiphospholipid antibody | Negative | - |
| **Anti-Ro** | **Positive** | **-** |
| APTT | 24.8 seconds | 20-30 seconds |
| Cardiolipin | Negative | - |
| Centromere Ab | Negative | - |
| **Complement C3** | **44.1 mg/dL** | **65-190 mg/dL** |
| **Complement C4** | **7.4 mg/dL** | **14-40 mg/dL** |
| **C-reactive protein** | **115 mg/L** | **<5 mg/L** |
| **dsDNA Ab** | **40 IU/mL** | **0-27 IU/mL** |
| **ENA Ab** | **Positive** | **-** |
| **ESR** | **34 mm/hr** | **0-30 mm/hr** |
| Gastric parietal cell Ab | Negative | - |
| IgA | 2.63 g/L | 0.8-3.0 g/L |
| **IgG** | **19.6 g/L** | **6-16 g/L** |
| IgM | 1.66 g/L | 0.4-2.5 g/L |
| INR | 1.0 | 0.8-1.2 |
| Jo-1 Ab | Negative | - |
| Liver and kidney microsomal Ab | Negative | - |
| Malaria Ag | Negative | - |
| Mitochondrial Ab | Negative | - |
| Rheumatoid factor | <7 IU/mL | 0-20 IU/mL |
| Scl-70 Ab | Negative | - |
| Smooth muscle IgG Ab | Negative | - |
| Thrombin time | 21 seconds | 14-21 seconds |
| Urine protein | 159 mg/L | - |
| Urine protein:creatinine ratio | 16.2 mg/mmol | - |
